# Divergence in gut bacterial community between females and males in the wolf spider *Pardosa astrigera*

**DOI:** 10.1002/ece3.8823

**Published:** 2022-04-12

**Authors:** Ying Gao, Pengfeng Wu, Shuyan Cui, Abid Ali, Guo Zheng

**Affiliations:** ^1^ 12402 College of Life Sciences Shenyang Normal University Shenyang China; ^2^ Department of Entomology University of Agriculture Faisalabad Pakistan

**Keywords:** Actinobacteriota, gut microbiota, *Rhodococcus*, sex, spider

## Abstract

Sex is one of the important factors affecting gut microbiota. As key predators in agroforestry ecosystem, many spider species show dramatically different activity habits and nutritional requirements between females and males. However, how sex affects gut microbiota of spiders remains unclear. Here, we compared the composition and diversity of gut bacteria between female and male *Pardosa astrigera* based on bacterial 16S rRNA gene sequencing. Results showed that the richness of bacterial microbiota in female spiders was significantly lower than in male spiders (*p* < .05). Besides, β‐diversity showed a significant difference between female and male spiders (*p* = .0270). The relative abundance of Actinobacteriota and *Rhodococcus* (belongs to Actinobacteriota) was significantly higher in female than in male spiders (*p* < .05), whereas the relative abundance of Firmicutes and *Acinetobacter* (belongs to Proteobacteria) and *Ruminococcus* and *Fusicatenibacter* (all belong to Firmicutes) was significantly higher in male than in female spiders (*p* < .05). The results also showed that amino acid and lipid metabolisms were significantly higher in female than in male spiders (*p* < .05), whereas glycan biosynthesis and metabolism were significantly higher in male than in female spiders (*p* < .05). Our results imply that sexual variation is a crucial factor in shaping gut bacterial community in *P*. *astrigera* spiders, while the distinct differences of bacterial composition are mainly due to their different nutritional and energy requirements.

## Graphical abstract

Sex is a crucial factor in shaping gut bacterial community in Pardosa astrigera. The male spiders disperse more widely than the females; hence, the males had a higher bacterial diversity, while the significant differences of bacterial composition are mainly due to their different nutritional and energy requirements.

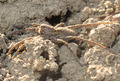

## 1 INTRODUCTION

Sex is an important factor influencing gut microbiota (Costello et al., 2012; Koren et al., 2012). Female and male insects exhibit different ecological behaviors in terms of nutritional and dispersal capabilities (Minard et al., 2013; Rani et al., 2009), which lead to different gut microbiota community in host. For instance, Foster (1995) and Zouache et al. (2011) showed that male mosquitoes dispersed less than the female, which could be a factor constraining bacterial diversity. Minard et al. (2013) also found that the different nutritional requirements between two sexes of mosquitos affected bacterial microbiota composition. Moreover, Wan et al. (2020) showed that higher gut bacterial diversity in females might contribute to the vertical transmission. Although dramatically different activity habits between the two sexes were shown in spider variable species, the effect of sex on gut microbiota of spiders was almost ignored.

Spiders are key predators in agroforestry ecosystem (Nyffeler & Birkhofer, 2017). The research on gut microbiota of spiders mainly focused on the following aspects. Hu et al. (2019) and Kumar et al. (2020) investigated the diversity and composition of gut microbiota from a few spider species and found that Proteobacteria was the most dominant phylum in Lycosidae, Titanoecidae, and Thomisidae, while Firmicutes was the most dominant phylum in Oxyopidae. Kennedy et al. (2020) suggested that the structure of gut microbiota in *Badumna longinqua* (Desidae) was dictated by the consumed prey; different prey taxa may remodel gut microbiota in different ways. Hu (2019) compared the tissue‐ and population‐level microbiota of spiders between ovaries and testicle, and the results showed that the relative abundance of most bacteria was significantly different, but the difference between the gut and gonad was not significant. Besides, the significant differences of microbiome were found between populations and individuals but not be found between tissue types (Sheffer et al., 2019). However, the effect of sex on gut microbiota of *Pardosa astrigera* has not been reported.

The wolf spider *Pardosa astrigera* L. Koch 1878 is a wandering spider which distributes widely throughout terrestrial environments, including agricultural lands in East Asia (World Spider Catalog, 2021). It is a very active ground‐dwelling predator and dominant species in most parts of China (Li et al., 2020). As a generalist predator, *P*. *astrigera* plays an important role in pest control in farmland ecosystem (e.g., as the natural enemy of *Plutella xylostella* [Plutellidae] on both cabbage and oilseed rape) (Quan et al., 2011). Obvious behavioral differences between female and male *P*. *astrigera* during the breeding period have been reported. That is, the female spiders usually adopt a “sit and wait” strategy, which avoids energy loss and being preyed by natural enemies, whereas the male is very active in searching for female everywhere (Chen & Song, 1999). Thus, *P*. *astrigera* is a good agent to investigate the effect of sex on gut microbiota of spiders. In this study, we investigated the gut bacterial community of female and male *P*. *astrigera* by high‐throughput sequencing. We hypothesized that (i) the diversity of gut bacteria in male *P*. *astrigera* would be higher than in female, and (ii) the effect of sex on dominant gut microbiota would be different, and the variations could be explained by the differences of metabolic function related to energy demand.

## 2 MATERIALS AND METHODS

### 2.1 Spider and sample collection

**FIGURE 1:**
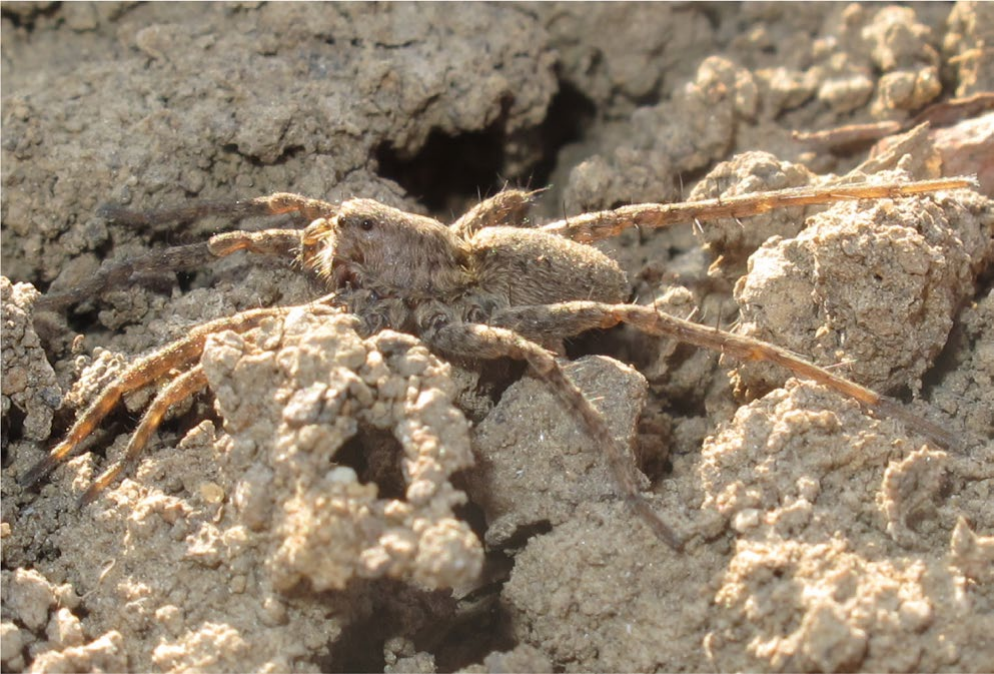
Photo of *Pardosa astrigera* in nature

Adult specimens of *P*. *astrigera* (female, *n* = 40; male, *n* = 40) were randomly collected by pipe buckle method on April 10, 2021 from cornfield (41°56′N, 123°22′E) aside by Puhe river, Northern Shenyang city, China (Figure 1). All spiders were transported to laboratory (Insect Ecology Laboratory, College of Life Sciences) and kept singly in plastic tubes (each 30 mm in diameter and 110 mm long) with moistened cotton at the bottom to maintain air humidity.

Spiders were starved for 10 days before dissection to remove the non‐native microorganisms in the gut (Hu et al., 2019). To ensure the sterile condition during dissection, the aseptic table was wiped with 75% ethanol for three times and irradiated with ultraviolet lamp for 60 min. Before dissection, each spider was sterilized by 75% ethanol for 5 min while rinsing three times by sterile water before and after sterilization to remove contaminants on its body surface. Then, the residual water on the surface of spider was sucked by sterilized filter paper. The gut was dissected in sterile phosphate‐buffered saline (PBS) solution with a sterilized scissor under microscope and washed with sterile water, placed into 1.5‐ml microcentrifuge tube, and temporarily stored in refrigerator (Haier BCD‐252WBCS, Qingdao, China) at −20°C. The process of dissection was finished on ice. Ten guts were added in a tube as one sample and instantly quick‐froze in liquid nitrogen, stored in a −80°C freezer (AUCMA DW‐86L500, Qingdao, China) until DNA extraction. Each female and male spider has four biological replicates, respectively.

### 2.2 DNA extraction and 16S rRNA gene amplicon sequencing

The total DNA of each pooled sample was extracted using the FastDNA Spin Kit for Soil (MP Biomedicals, USA) following the manufacturer's protocol. The quality and integrity of collected DNA were assessed by 1% agarose gel electrophoresis; its concentrations and purities were determined with a NanoDrop 2000 spectrophotometer (Thermo Fisher Scientific, Wilmington, USA).

The DNA was amplified using 16S rRNA gene V3‐V4 region primers 338F (5′‐ACTCCTACGGGAGGCAGCAG‐3′) and 806R (5′‐GGACTACHVGGGTWTCTAAT‐3′) (Kumar et al., 2020). The Polymerase Chain Reaction (PCR) amplification contains 4‐μl 5 × buffer, 2‐μl dNTPs (2.5 mM), 0.8‐μl forward primer (5 μM), 0.8‐μl reverse primer (5 μM), 0.4‐μl DNA polymerase, 10‐ng template DNA, and finally ddH_2_O up to 20 μl. The PCR reaction under the following conditions: initial denaturation at 95°C for 3 min, and 29 cycles of denaturation at 95°C for 30 s, annealing at 53°C for 30 s and extension at 72°C for 45 s, and a final extension at 72°C for 10 min. The PCR product was extracted from 2% agarose gel and purified using the AxyPrep DNA Gel Extraction Kit (Axygen Biosciences, Union City, CA, USA) according to manufacturer's instructions and quantified using Quantus™ Fluorometer (Promega, USA). Sequencing was carried out on an Illumina MiSeq platform at Majorbio Bio‐Pharm Technology Co., Ltd., Shanghai, China.

### 2.3 Bioinformatics, sequence analysis, and statistical analysis

In order to obtain more reliable and high‐quality sequencing results (valid reads), the following pre‐procedures were performed on the raw reads from the Illumina MiSeq platform: raw reads were demultiplexed, quality filtered by FASTP (version 0.19.6; Chen et al., 2018), and merged by FLASH (version 1.2.11; Magoc & Salzberg, 2011), and high‐quality reads were clustered as an operational taxonomic unit (OTU) by UPARSE (version 7.0) when the sets of sequences shared at least 97% identity (Edgar, 2013), and chimeric sequences were identified and removed. All OTUs with totaling reads more than 50 were used. The taxonomy of each OTU representative sequence was analyzed by RDP Classifier (version 2.11; Wang et al., 2007) against the Silva 16S rRNA database (version 138) using confidence threshold of 70% (Quast et al., 2013).

Mothur software (version 1.30.2) was employed to calculate α‐diversity including Sobs, Chao1, Shannon, Simpson, and Coverage. Student's *t*‐test was performed to compare α‐diversity estimates; *p*‐value less than .05 was considered significant in statistics. β‐diversity analysis was performed and visualized using principal coordinate analysis (PCoA) based on Bray–Curtis distances calculated from OTU compositions. In addition, a permutation multivariate analysis of variance (Adonis test with 999 permutations) was performed to test for the effects of sex on microbial community structures.

Taxa abundances in two sexes at the phylum and genus levels were compared by Wilcoxon rank‐sum tests and two‐tailed *p*‐value less than .05 was considered significant (with bootstrap values 95%). The different biomarkers associated with sex were characterized by linear discriminant analysis (LDA) effect size (LEfSe) (Segata et al., 2011). Microbial functions were predicted by using phylogenetic investigation of communities by reconstruction of unobserved states 2 (PICRUSt2) based on high‐quality sequences. Independent sample *t*‐test was further performed to test whether the difference between the two sexes was significant. All statistical analyses were conducted using SPSS statistical software (SPSS, version 26.0), and the diagrams were finished by Origin software (version 2019).

## 3 RESULTS

### 3.1 Bacterial 16S rRNA sequence data

A total of 335455 raw reads were generated from eight samples. After quality filtering, 299149 valid reads were obtained while remaining with an average of 37393 valid reads per sample. All estimated coverage values were over 99% which indicated that current sequences sufficiently covered the diversity of the sample of bacterial communities (Table 1). About 268 OTUs, clustered at 97% sequence similarity, were detected in all samples. Among them, 155 OTUs were shared between both sexes; 110 OTUs were specific in male samples, whereas only three OTUs were specific in female samples.

**TABLE 1:** The α‐diversity indices (Mean ± SD) of bacterial communities of *Pardosa astrigera*. The differences based on Student's *t*‐test

	Sobs	Chao1	Shannon	Simpson	Coverage
Female	91.25 ± 15.63	92.28 ± 17.09	0.96 ± 0.27	0.71 ± 0.09	0.9999
Male	153.75 ± 39.98	159.03 ± 40.98	2.96 ± 0.38	0.16 ± 0.03	0.9998
*p*‐value	.0269	.0238	.0001	.0000	.1135

Note

*p* < .05 indicates significant difference.

### 3.2 Bacterial diversity between the two sexes

Bacterial community richness and diversity varied between female and male *P*. *astrigera* (Table 1). The results of Student's *t*‐test showed that bacterial α‐diversity of Sobs (*p* = .0269), Chao1 (*p* = .0238), and Shannon index (*p* = .0001) in females was significantly lower than in males, whereas Simpson index (*p* = .0000) in females was significantly higher than in males. Meanwhile, males showed a much larger standard deviation in Sobs and Chao1 indices than females.

**FIGURE 2:**
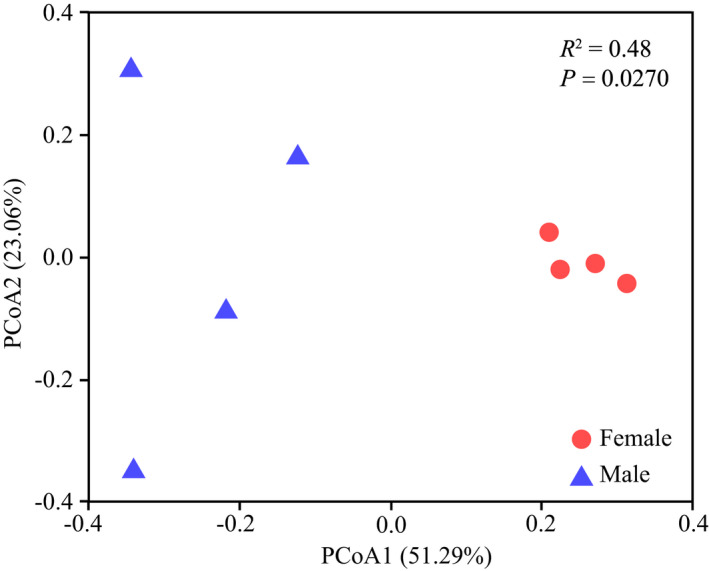
Beta diversity difference in gut bacteria within sex of *Pardosa astrigera*. Principal coordinate analysis (PCoA) based on Bray–Curtis distances and Adonis test (with 999 permutations) to show differentiation in microbial structures of different sexes

The PCoA results of β‐diversity illustrated that bacterial community of the two sexes clustered separately. The results of Adonis test showed significant difference between females and males (*p* = .0270, *R* ^2^ = .48; Figure 2) in their community composition and relative abundance, and the variation range among male samples was much greater than that of female samples.

### 3.3 Bacterial compositions between the two sexes

**FIGURE 3:**
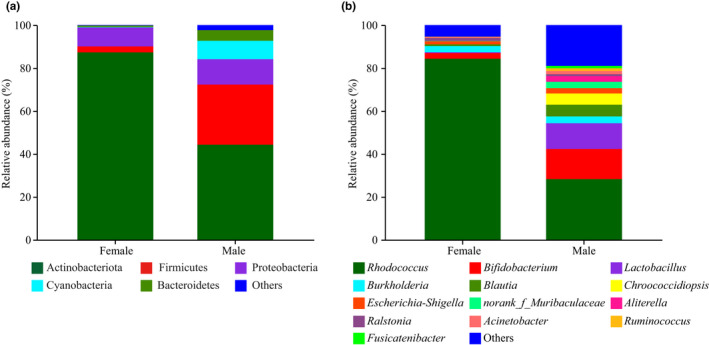
Gut bacterial compositions at the level of phylum (a) and genus (b) from *Pardosa astrigera*. Taxa with less than 1% membership in samples of each group are grouped within “Others”

**FIGURE 4:**
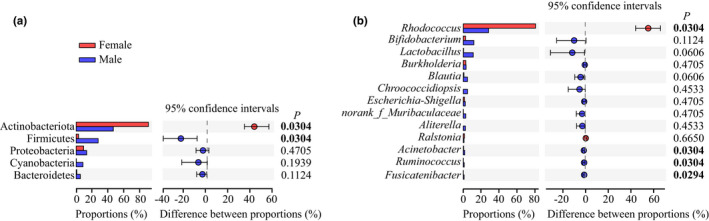
The gut bacterial composition and difference at the level of phylum (a) and genus (b) in sex from *Pardosa astrigera*. The difference based on Wilcoxon rank‐sum test and a two‐tailed *p*‐value less than .05 was considered significant (with bootstrap values 95%)

The relative abundances of dominant (>1%) gut bacteria showed evident differences between two sexes at different taxon levels (Figure 3). At phylum level, a total of 21 phyla were identified across all data, among these, Actinobacteriota, Firmicutes, and Proteobacteria were the dominant phyla in both sexes, and Cyanobacteria and Bacteroidetes were dominant in male spiders (Figure 3a). The results of Wilcoxon rank‐sum test indicated that females had a significantly higher relative abundance of Actinobacteriota (*p* = .0304) and a significantly lower relative abundance of Firmicutes (*p* = .0304) relative to males (Figure 4a). Other dominant phyla were all higher in male than in female spiders, though no significant difference was observed. At genus level, a total of 168 genera were found across all data, among these, 5 dominant genera belong to females, whereas 12 dominant genera belong to males (Figure 3b). The results of Wilcoxon rank‐sum test showed that females had a significantly higher relative abundance of *Rhodococcus* (*p* = .0304) and a significantly lower relative abundance of *Acinetobacter* (*p* = .0304), *Ruminococcus* (*p* = .0304), and *Fusicatenibacter* (*p* = .0294) relative to males (Figure 4b). Other dominant genera had no significant difference between the two sexes.

**FIGURE 5:**
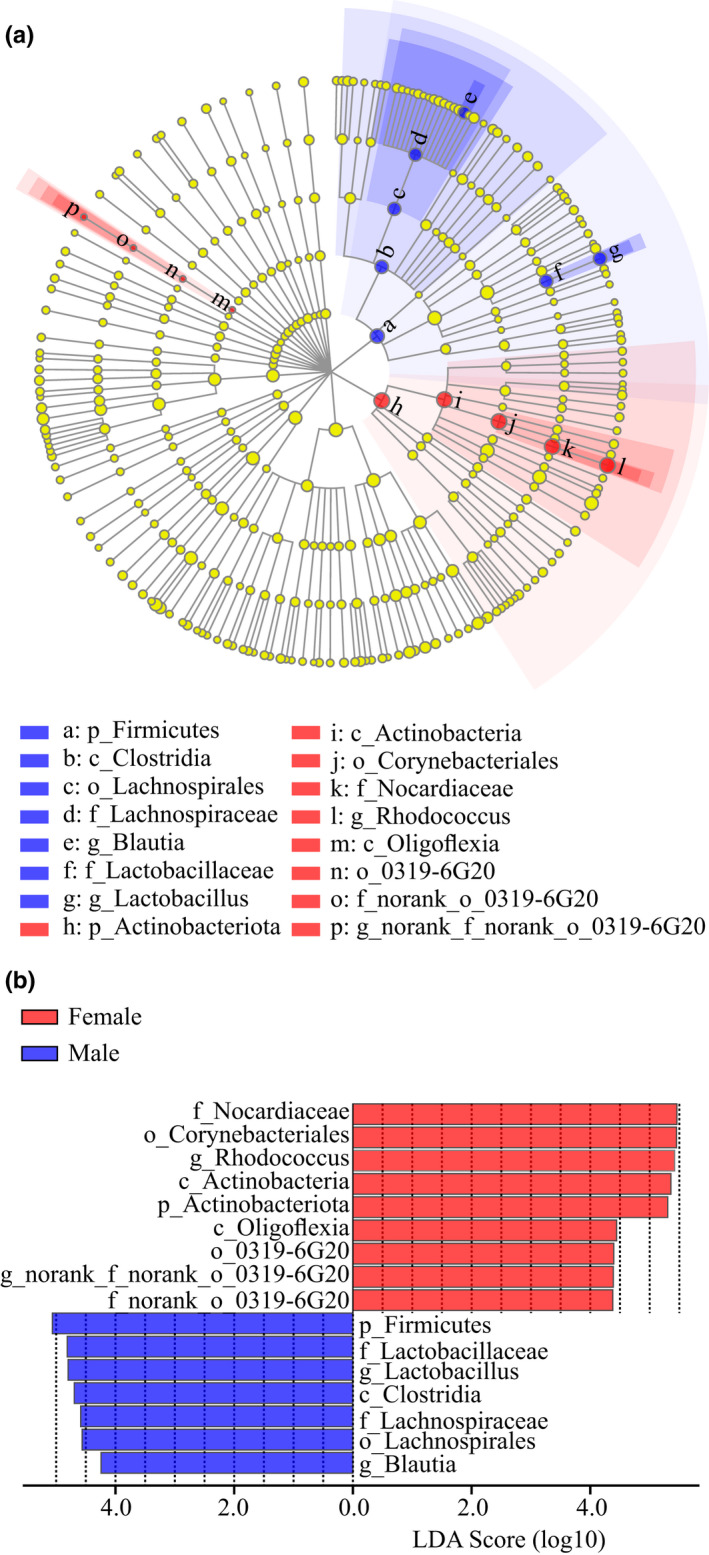
LEfSe analysis for remarking the significantly abundant bacterial community of *Pardosa astrigera*. (a) Cladogram showing the relationship among taxa (from the inner to outer rings, phylum, class, order, family, and genus). (b) The bar plot showing the different taxa with a LDA score >4, *p* < .05

In agreement with community composition, noteworthy divergences in bacterial community were found from the result of LEfSe analysis between female and male spiders, based on relative abundance of biomarkers of bacteria. Two groups of bacteria, namely *Rhodococcus* (from phylum to genus) and *norank_f_norank_o_0319*‐*6G20* (from class to genus), were significantly enriched in female spiders, whereas *Blautia* (from phylum to genus) and *Lactobacillus* (the phylum and family to genus) were significantly enriched in male spiders (LDA Score >4, *p* < .05; Figure 5a, b).

### 3.4 Functional predictions with PICRUSt2

**FIGURE 6:**
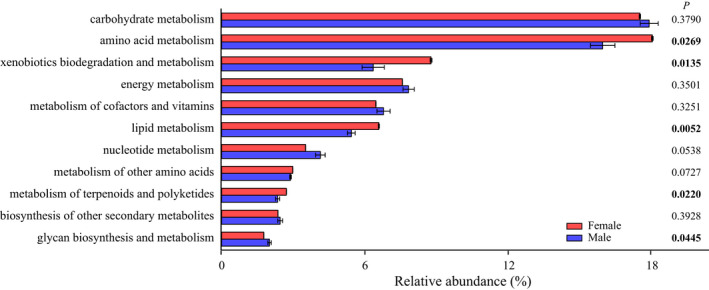
Comparison of predicted function of gut bacteria in *Pardosa astrigera*. The difference based on independent sample *t*‐test

A total of 11 level‐2 pathways associated with key metabolic functions were contained in the result of functional predictions using PICRUSt2 analysis. Among these, amino acid metabolism (*p* = .0269), xenobiotics biodegradation and metabolism (*p* = .0135), lipid metabolism (*p* = .0052), and metabolism of terpenoids and polyketides (*p* = .0220) in females were significantly higher than males, whereas glycan biosynthesis and metabolism (*p* = .0445) in females was significantly lower than in males (Figure 6).

## 4 DISCUSSION

This study compared the gut bacterial community between female and male *P*. *astrigera*. Although all individual spiders used in the present study were collected at early spring season from a highly homogeneous cornfield in a very small range, obvious differences in gut bacterial diversity and composition were found between the two sexes. Our results prove that sexual variation is a crucial factor in shaping the gut bacterial community in spiders.

Contrary to the previous results that higher gut bacterial diversity was found in female insects (Han et al., 2017; Mason et al., 2019; Wan et al., 2020; Xu et al., 2016), we found that male spiders had higher gut bacterial richness than female spiders. Environmental factors have been proved to be important roles in gut microbiota assembly in arthropods (Chandler et al., 2011; Wong et al., 2013); insects can obtain microbiota from their surrounding environments (Douglas, 2011). For the reason that female *P*. *astrigera* takes a “sit and wait” strategy during breeding period, the male may wander around for seeking female spiders, thus male spiders have more environmental exposure. Consistent with our first hypothesis, different sex‐related behaviors result in a significantly higher bacterial richness in male spiders than in female spiders. This finding corresponds with those of Foster (1995) and Zouache et al. (2011), which suggested that less dispersal and more retention around breeding sites could be factors constraining bacterial diversity of male mosquitoes. Similarly, Ng et al. (2018) reported that reduced constant environmental exposure could decrease gut bacterial diversity in crickets.

Our results also showed that significant differences in gut bacterial community composition were found between female and male *P*. *astrigera*, and the relative abundance of dominant bacteria differed in different taxon level between the female and male. The differences of gut bacterial community composition are mainly caused by various metabolic activities induced by different nutritional needs. Female spiders try to avoid the reduction of energy and accumulate substantial nutrients for spawning simultaneously. As a result, very high Actinobacteriota and *Rhodococcus* (belongs to Actinobacteriota) were found in female spiders, which probably due to the reason that Actinobacteriota needs nutritional supplement for normal growth (Salem et al., 2013). On the contrary, we found that male spiders have higher Firmicutes and Firmicutes/Bacteroidetes (F/B) ratio than female spiders, which contribute to the decomposition of complex carbohydrates, fatty acids, polysaccharides (Flint et al., 2008), and energy harvest (Ng et al., 2018; Turnbaugh et al., 2006; Yu et al., 2020). Therefore, high Firmicutes and F/B ratio might meet the energy needs of male spiders which wander in the breeding period for seeking the female spiders. We also note that bacterial genera unrelated to sex were similar between female and male *P*. *astrigera*. For example, fenitrothion‐resistant *Burkholderia* has ability to hydrolyze the compound, thus protect its host (Kikuchi et al., 2011).

Our results demonstrated the effect of sex on gut bacteria of spiders. Specially, *P*. *astrigera* spider has significant differences in gut bacteria due to different behavior and physiological needs. Male *P*. *astrigera* has been habituated to wander around for seeking female spiders, thus has a significantly higher gut bacterial richness than female which has “sit and wait” strategy. Moreover, the female has a high relative abundance of Actinobacteriota which helps to meet their need of spawning and reproduction, whereas the male has high relative abundance of Firmicutes and F/B ratio due to their energy demand of searching for partners. In conclusion, sexual dimorphism is a very common phenomenon; the potential importance of sex on gut bacteria should not be ignored in future research in spiders.

## CONFLICT OF INTEREST

The authors declare no conflicts of interest.

## AUTHOR CONTRIBUTIONS

**Ying Gao:** Data curation (equal); Investigation (equal); Writing – original draft (equal). **Pengfeng Wu:** Data curation (equal); Project administration (equal); Writing – review & editing (equal). **Shuyan Cui:** Writing – review & editing (equal). **Abid Ali:** Writing – review & editing (equal). **Guo Zheng:** Data curation (equal); Funding acquisition (equal); Supervision (equal); Writing – original draft (equal).

## Data Availability

The original data of the gut microbiota relative abundance in spiders are available from the NCBI Sequence Read Archive (SRA) database (Accession number: PRJNA781009).
